# The Impact of COVID-19 on the Epidemiology of Carbapenem Resistance

**DOI:** 10.3390/antibiotics14090916

**Published:** 2025-09-11

**Authors:** Aikaterini Sakagianni, Christina Koufopoulou, Petros Koufopoulos, Georgios Feretzakis, Vasiliki Koumaki

**Affiliations:** 1 Intensive Care Unit, Sismanogleio General Hospital, 15126 Marousi, Greece; 2 Anesthesiology Department, General Hospital of Athens “G. Gennimatas”, 11527 Athens, Greece; ckoufopoulou@med.uoa.gr; 3 Internal Medicine Department, Sismanogleio General Hospital, 15126 Marousi, Greece; petroskouf@med.uoa.gr; 4 School of Science and Technology, Hellenic Open University, 26131 Patras, Greece; georgios.feretzakis@ac.eap.gr; 5 Department of Microbiology, Medical School, National and Kapodistrian University of Athens, 11527 Athens, Greece; vkoumaki@med.uoa.gr

**Keywords:** carbapenem resistance, antimicrobial resistance, COVID-19, infection control, AMR surveillance

## Abstract

**Background**: The global COVID-19 pandemic has significantly disrupted healthcare systems, inadvertently influencing the epidemiology of antimicrobial resistance (AMR). Among the most critical AMR threats are carbapenem-resistant organisms (CROs), which include carbapenem-resistant *Enterobacterales*, *Acinetobacter baumannii*, and *Pseudomonas aeruginosa*. This review explores the pandemic’s impact on carbapenem resistance patterns worldwide. **Objectives**: This study aimed to assess the effects of the COVID-19 pandemic on carbapenem resistance trends, identify key drivers, and discuss implications for clinical practice and public health policy. **Methods**: A comprehensive review of peer-reviewed literature, national surveillance reports, and WHO/ECDC data from 2019 to 2025 was conducted, with emphasis on hospital-acquired infections, antimicrobial use, and infection control practices during the pandemic. **Results**: The pandemic has led to increased use of broad-spectrum antibiotics, including carbapenems, often in the absence of confirmed bacterial co-infections. Overwhelmed healthcare systems and disruptions in infection prevention and control (IPC) measures have facilitated the spread of carbapenem-resistant organisms, particularly in intensive care settings. Surveillance data from multiple countries show a measurable increase in CRO prevalence during the pandemic period, with regional variations depending on healthcare capacity and stewardship infrastructure. **Conclusions**: COVID-19 has accelerated the emergence and dissemination of carbapenem resistance, underscoring the need for resilient antimicrobial stewardship and IPC programs even during public health emergencies. Integrating pandemic preparedness with AMR mitigation strategies is critical for preventing further escalation of resistance.

## 1. Introduction

The global rise in AMR poses a critical threat to public health, with carbapenem-resistant organisms ranking among the most challenging to treat. Carbapenems are broad-spectrum β-lactam antibiotics used as escalation therapies, particularly against multidrug-resistant Gram-negative bacteria such as *Klebsiella pneumoniae*, *Acinetobacter baumannii*, and *Pseudomonas aeruginosa*. While they are not the absolute last line of treatment, carbapenems are reserved for infections resistant to first-line therapies [1]. The emergence and proliferation of carbapenem-resistant strains—particularly carbapenem-resistant *Enterobacterales* (CRE), carbapenem-resistant *Acinetobacter baumannii* (CRAB), and carbapenem-resistant *Pseudomonas aeruginosa* (CRPA)—have led to increased morbidity, mortality, and healthcare costs worldwide [2,3].

The COVID-19 pandemic, caused by the novel coronavirus SARS-CoV-2, introduced unexpected challenges to healthcare systems globally. While the primary focus was on managing viral transmission and acute respiratory illness, the pandemic also exerted substantial indirect effects on antimicrobial usage patterns, infection control practices, and resistance surveillance efforts [4,5]. Hospitals were overwhelmed, antimicrobial stewardship programs (ASPs) were disrupted, and empirical antibiotic prescribing increased dramatically—even in cases where bacterial co-infection was unlikely [6,7].

Several reports during the pandemic indicated rising trends in the incidence of carbapenem-resistant infections, particularly in intensive care settings [8,9]. Simultaneously, gaps in routine infection prevention protocols, coupled with heavy reliance on invasive interventions such as mechanical ventilation, may have contributed to the accelerated spread of resistant organisms within hospitals [10,11].

This review aims to integrate current evidence on the impact of the COVID-19 pandemic on the epidemiology of carbapenem resistance. We examine how changes in clinical practice, antimicrobial prescribing, and infection control contributed to shifts in resistance patterns. By exploring surveillance data, resistance mechanisms, and treatment challenges, this article also offers insights into how healthcare systems can better prepare for future public health emergencies without compromising the fight against AMR.

## 2. Antimicrobial Use During the COVID-19 Pandemic

At the onset of the COVID-19 pandemic, clinicians frequently prescribed antibiotics to patients with suspected or confirmed SARS-CoV-2 infection, driven by concerns over possible bacterial co-infection. Early studies reported bacterial co-infection rates of only 7–15% among hospitalized COVID-19 patients, yet antibiotic prescribing rates exceeded 70% in some settings [12,13]. Carbapenems and other broad-spectrum agents were commonly used, particularly in intensive care units (ICUs), where patients were critically ill and often required invasive mechanical ventilation [14].

This widespread use was largely empirical, stemming from diagnostic uncertainty and the urgency to prevent secondary infections in immunocompromised individuals. Furthermore, the lack of rapid diagnostics for distinguishing viral from bacterial infections added to the over-reliance on antibiotics [4,5].

Several observational studies reported increased consumption of carbapenems during peak COVID-19 waves. For example, hospitals in India and Italy saw significant spikes in meropenem usage compared to pre-pandemic levels [15,16]. These trends were mirrored in multiple countries, raising alarms about the unintended consequence of promoting carbapenem resistance.

In addition, the disruption of ASPs during the pandemic contributed to inappropriate prescribing practices. Many ASPs were deprioritized or suspended due to the reallocation of healthcare personnel to COVID-19 care [17,18,19]. Without active monitoring and feedback, clinicians lacked guidance on optimal antibiotic use, further amplifying the problem.

The overuse of antibiotics during the pandemic created selective pressure favoring the proliferation of resistant pathogens, including carbapenemase-producing organisms [20,21]. This trend underscores the critical need for pandemic preparedness plans that integrate robust antimicrobial stewardship even in crisis scenarios.

## 3. Infection Prevention and Control Challenges

The COVID-19 pandemic significantly disrupted infection prevention and control (IPC) practices in healthcare settings worldwide. These disruptions created favorable conditions for the spread of carbapenem-resistant organisms, particularly in ICUs and COVID-19 wards.

### 3.1. Resource Reallocation and Staff Shortages

As hospitals prioritized COVID-19 response efforts, human and material resources were diverted from established IPC programs [11]. Many facilities experienced critical shortages of trained infection control personnel, and routine surveillance for multidrug-resistant organisms (MDROs) was scaled back or suspended during the pandemic’s peak periods [22]. Staff relocation and fatigue further compromised adherence to IPC guidelines, including hand hygiene and environmental cleaning protocols.

### 3.2. Overcrowding and Invasive Procedures

The surge of critically ill COVID-19 patients led to overcrowded ICUs and increased use of invasive devices such as ventilators, central lines, and urinary catheters—well-known risk factors for healthcare-associated infections (HAIs) and colonization by resistant pathogens [23]. These interventions created more opportunities for cross-transmission, especially when isolation precautions were inconsistently implemented due to space constraints or emergency conditions. A genomic study in a COVID-19 ICU in Italy, demonstrated that 35% of ventilated patients were colonized and 65% infected with CRAB; transmission chains were traced to shared ICU modules and breathing circuits. The study highlights how overwhelmed ICU conditions and shared medical equipment during the COVID-19 pandemic facilitated the transmission of AMR, underscoring the urgent need for reinforced infection control measures during health crises [24].

### 3.3. PPE Shortages and Misuse

Shortages of personal protective equipment (PPE), particularly early in the pandemic, forced many institutions to adopt crisis standards of care, including PPE reuse and extended use [25]. Although necessary under emergency conditions, these practices may have inadvertently increased the risk of MDRO transmission between patients. Studies also reported improper donning and doffing practices among reallocated or undertrained healthcare workers, creating contamination risks during PPE removal [26,27]. A 2022 audit in South Korea found that despite enhanced COVID-19 precautions, an outbreak of CRAB occurred in a COVID-19 ward—resolved only after enhanced environmental cleaning and additional gowning/gloving measures were implemented [28].

### 3.4. Reduced Focus on Antimicrobial Stewardship and IPC Monitoring

Routine audits, feedback, and training sessions for IPC and antimicrobial stewardship were deprioritized or halted during peak COVID-19 periods [4]. A nationwide survey of antimicrobial stewardship pharmacists in the UK reported that 65% of facilities perceived a negative impact on routine AMS activities, including multidisciplinary ward rounds and point prevalence surveys, with 31% reporting a “very negative” effect [29]. This reduction in oversight led to an increased risk of guideline deviations and contributed to the unhindered spread of carbapenem-resistant bacteria within hospitals. Likewise, a national survey of acute-care pharmacists in the U.S. found a marked shift away from direct patient care and education toward administrative and pandemic response duties, leading to increased prescribing errors and reduced stewardship interventions [30]. In hospitals where ASPs remained operational, prospective audit and feedback interventions significantly reduced unnecessary antimicrobial use in COVID-19 patients—demonstrating the value of maintaining stewardship even during crises [31,32].

### 3.5. Documented CRO Outbreaks Linked to IPC Disruption

Several outbreaks of carbapenem-resistant *Enterobacterales* and *Acinetobacter baumannii* have been linked directly to IPC lapses in COVID-19 care units. During the COVID-19 surge in New Jersey, hospital-acquired cases of CRAB significantly increased, reflecting disrupted infection control strategies and increased patient load [33]. In Germany, case–control analysis of three CRAB outbreaks highlighted ICU overcrowding and medical procedures as independent risk factors, underscoring insufficient IPC measures amid pandemic surge [34]. Similar outbreaks of CRAB were documented globally during the COVID-19 pandemic, underscoring the widespread vulnerability of healthcare settings [10,28,35,36].

## 4. Surveillance Data and Epidemiological Trends

The COVID-19 pandemic disrupted AMR surveillance efforts worldwide. Despite this, emerging data from national and international healthcare systems have documented concerning increases in the prevalence of carbapenem-resistant organisms during and following major waves of COVID-19.

### 4.1. Global Trends and Surveillance Reports

In the United States, the Centers for Disease Control and Prevention (CDC) reported a significant increase in HAIs involving multidrug-resistant pathogens during the COVID-19 pandemic [8]. Notably, infections caused by CRAB rose by 78% between 2019 and 2020, representing the largest relative increase among tracked antimicrobial-resistant organisms [8].

Carbapenem-resistant *Enterobacterales* (CRE) also rebounded after several years of decline. While national trends had previously shown a steady decrease due to strengthened infection prevention and antimicrobial stewardship programs, CRE rates surged by 35% in 2020, with the most pronounced rise—a 60% increase—occurring between first and third quarter of that year [8]. This escalation coincided with COVID-19 patient surges and was attributed to factors such as prolonged ICU stays, overuse of broad-spectrum antibiotics, and systemic strain on infection control protocols.

Importantly, these increases were not transient. A CDC analysis, the second year of the pandemic (2021) found continued elevation in HAIs [37]. Incidence rates of CRE and CRAB remained significantly higher than pre-pandemic levels, underscoring persistent challenges in infection prevention as hospitals continued to cope with patient surges, staff shortages, and resource constraints. These findings underscore the sustained collateral impact of the pandemic on antimicrobial resistance and highlight the urgency of rebuilding resilient surveillance and infection prevention systems.

Similarly, the European Centre for Disease Prevention and Control (ECDC) reported that in 2021, over one-third of EU/EEA countries had ≥25% of *Klebsiella pneumoniae* isolates resistant to carbapenems, with even higher resistance rates observed for *Acinetobacter species* in several regions [9]. A marked resurgence of carbapenemase-producing *K. pneumoniae* and *Pseudomonas aeruginosa* was documented particularly in southern and eastern Europe. The trend reversed from pre-2019 slow decline or stabilization to sharp increases during the COVID-19 pandemic, with sustained high levels of CRE reported through 2024–2025. Bloodstream infections caused by high-risk and increasingly hypervirulent *K. pneumoniae* clones—such as ST23—were reported across 23 EU/EEA countries [20].

For CRAB, ECDC surveillance via EARS-Net revealed wide variation across the EU, ranging from <1% to ≥50% of invasive *Acinetobacter* isolates in 2021, with the highest resistance levels reported in Southern and eastern Europe [9]. A pronounced surge in CRAB bloodstream infections was observed between 2020 and 2021, coinciding with healthcare system disruptions during the COVID-19 crisis [38]. This shift highlights an urgent need for enhanced regional preparedness, reinforced surveillance systems, and strengthened IPC strategies [9,20].

#### Insights from Emerging Economies and Global Surveillance

The impact of the COVID-19 pandemic on carbapenem resistance was particularly pronounced in low- and middle-income countries (LMICs), where structural vulnerabilities and resource shortages in healthcare systems amplified the crisis [39,40]. Surveillance data from the ATLAS program (2018–2022) revealed consistently higher rates of CRE and CRAB in the Asia–Pacific (APAC), Latin America (LATAM), and Middle East–Africa (MEA) regions compared with North America and Europe, with prevalence rising further during the pandemic. CRE exceeded ~10% in APAC, LATAM, and MEA, while CRAB often surpassed 50% [41]. Similarly, WHO’s Global Antimicrobial Resistance and Use Surveillance System (GLASS) reported sharp post-2020 increases in CRE and CRAB across South Asia, the Middle East, and sub-Saharan Africa, largely driven by ICU-associated infections [42].

These findings highlight that, beyond established high-burden settings in Europe, emerging economies experienced disproportionate increases in carbapenem resistance during COVID-19. Pandemic-related factors—including antibiotic overuse, prolonged hospitalizations, and breakdowns in IPC—were key drivers. Notably, “difficult-to-treat resistance” (DTR) phenotypes, defined as nonsusceptibility to all first-line, high-efficacy agents, rose across all regions, underscoring the escalating therapeutic challenges [41]. In addition, early reports of community-onset CRE in APAC, LATAM, and MEA suggest a shift in transmission dynamics that contrasts with the hospital-dominated epidemiology typical of North America and Europe.

Parallel trends were observed for *Pseudomonas aeruginosa*. Li et al. [43] showed that while CRPA remained relatively stable in high-income countries, emerging economies saw disproportionate increases in both carbapenem-resistant and DTR phenotypes during COVID-19 surges—largely fueled by extensive broad-spectrum antibiotic use and limited access to novel agents. Similarly, Arshad et al. [39] emphasized that pandemic-driven antibiotic misuse, inadequate stewardship, and weakened IPC programs in developing regions created conditions for accelerated antimicrobial resistance, compounding pre-existing vulnerabilities.

Collectively, these findings suggest that emerging economies experienced disproportionate increases in carbapenem resistance during the pandemic, leading to a widening of global AMR burden and complicating both infection control and treatment strategies. The heterogeneity of these impacts is best appreciated in national and center-specific reports, which provide finer detail on regional trends.

### 4.2. Regional Observations

Beyond global surveillance, country- and center-specific reports illustrate the heterogeneity of carbapenem resistance trends during the COVID-19 pandemic. Germany experienced dynamic trends in carbapenem-resistant infections. Baum et al. reported that notifications of carbapenem-resistant pathogens in Germany dropped sharply during the first two years of the pandemic, with CRAB cases falling by about 30% in 2020 and 23% in 2021, alongside marked declines in CREs. These reductions were consistent across both statutory and laboratory-based surveillance systems and likely reflected the combined effects of fewer hospitalizations and reduced international mobility. By 2022, however, CRAB notifications exceeded projections by over 30%, with CRE also rebounding. The findings suggest that while the pandemic temporarily suppressed AMR transmission, the resurgence observed in 2022 may be linked to the resumption of hospital activities and increased global travel [44].

A multicenter Italian study involving 11,063 patients reported a significant increase in CRE colonization during the first year of the COVID-19 pandemic, particularly in Southern regions. Colonization at admission rose from 3.9% to 11.5%, while in-hospital acquisition increased from 5.1% to 15.5%. KPC-producing *Klebsiella pneumoniae* was the predominant strain identified. The findings underscore the importance of active surveillance and center-specific infection control strategies to limit CRE spread [45]. A retrospective study from a teaching hospital in Italy illustrates the limits of infection control during the COVID-19 pandemic. Despite a robust ICU-based ASP and active CRE surveillance, the conversion of the ICU to a COVID-19 unit in March 2020 led to a surge in CRE acquisition from 6.7% in 2019 to 50% in March–April 2020. Contributing factors included high-intensity care, frequent prone maneuvers requiring multiple staff involvement, and redeployed personnel lacking ICU experience [46]. This highlights how pandemic pressures can overwhelm established stewardship and infection control measures, emphasizing the need for adaptive strategies in high-burden settings.

Analyses of data from WHONET-Greece, covering January 2018 to March 2021, demonstrate significant pandemic-linked changes in resistance patterns. ICU *Acinetobacter baumannii* bloodstream isolates maintained near-universal meropenem non-susceptibility (96.6% in Q1 2018 to 100% in Q1 2021), while *Klebsiella pneumoniae* isolates exhibited abrupt increases in non-susceptibility during early pandemic (e.g., meropenem: +21.6%, imipenem: +20.8%, levofloxacin: +19.5%, all *p* = 0.001), which then remained elevated [47,48]. Surveillance from 2022 revealed the endemic spread of high-risk carbapenemase-producing *K. pneumoniae* clones (ST39, ST323, ST258/512) across 13 of 15 hospitals, with substantial intra-hospital transmission events (44 confirmed) indicating persistent spread despite fewer isolates compared to 2019 [49,50].

Recent surveillance from India further highlights the scale of the challenge in low- and middle-income settings. During the second wave of COVID-19, CRE and CRAB rates exceeded 60% in several ICUs, with many *K. pneumoniae* isolates co-harboring New Dehli metallo-beta-lactamase (NDM) and OXA-48 [51]. More recent surveillance from a South Indian tertiary care center confirmed this alarming trend, with 24% of Gram-negative isolates showing carbapenem resistance, predominantly in *Acinetobacter baumannii* (48.0%) and *K. pneumoniae* (38.6%). Resistance was particularly high among elderly male patients and in surgical wards, with a sharp peak in early 2023, where *A. baumannii* resistance reached 76.3%. Molecular profiling revealed the predominance of blaNDM and blaVIM genes across resistant organisms, underscoring the growing complexity of resistance mechanisms in high-risk clinical settings [52].

In Brazil, as an illustrative example from South America, multicenter surveillance across eight hospitals revealed significant increases in carbapenem-resistant infections during COVID-19 surges, with resistance levels remaining elevated even post-surge [14]. Markedly, resistance in *Klebsiella pneumoniae* doubled during the surge and persisted at high levels afterward, while *Pseudomonas aeruginosa* resistance showed a decline in the post-surge period. Molecular analysis confirmed high prevalence of KPC, NDM, and OXA-48 genes among ICU isolates. A regional study confirmed persistent CRE, with blaKPC in 78% and blaNDM in 22% of isolates, and a rising blaNDM trend linked to annual increases in COVID-19 cases [53]. Complementing these findings, a comprehensive seven-year national surveillance study (2015–2022) demonstrated that pandemic years were associated with >60% increases in plasmid-mediated carbapenemases among *Enterobacterales*, *P. aeruginosa*, and *Acinetobacter*. In particular, blaNDM exhibited sharp post-2020 increases, suggesting the pandemic accelerated its dissemination at the national level [54].

These regional reports reinforce that pandemic-associated resistance trends were not uniform. In summary, the COVID-19 pandemic has reshaped carbapenem resistance patterns globally, with regional surges reflecting both systemic pressures and lapses in infection control. These epidemiological changes underscore the need to examine the underlying mechanisms—enzymatic, structural, and environmental—that drive resistance, which will be addressed in the next section.

## 5. Mechanisms of Resistance Observed During the Pandemic

The epidemiological shifts in carbapenem-resistant infections described in Section 4 are closely intertwined with underlying molecular and functional mechanisms of resistance. Carbapenem resistance in Gram-negative bacteria is primarily mediated through enzymatic degradation via carbapenemases, modifications in membrane permeability, efflux pump overexpression, and biofilm formation, all of which contribute to persistence in healthcare environments [55]. Additionally, mobile genetic elements (MGEs) facilitated rapid horizontal gene transfer, amplifying the spread of high-risk clones within hospitals [56]. Understanding these mechanisms provides critical context for interpreting observed trends in the prevalence of CROs and highlights the multifaceted challenges posed by resistance during and after the pandemic.

### 5.1. Shifts in Carbapenemase Profiles

During the pandemic, resistance in Gram-negative bacteria was primarily driven by enzymatic degradation through carbapenemases such as KPC (Class A), NDM/VIM/IMP (Class B), and OXA-48-like (Class D) [45,51,52,53,54,55,57]. Reports from ECDC and CDC noted increased isolation of *Klebsiella pneumoniae* and *Acinetobacter baumannii* harboring these enzymes, often in combination [8,20]. These shifts reflect the selective pressure exerted by high antibiotic consumption, weakened antimicrobial stewardship, and disruptions to IPC programs. For example, a recent retrospective study conducted in a tertiary hospital in Greece, analyzed 2021 non-duplicate carbapenemase-producing *Enterobacterales* (CPE) isolates over a four-year period, encompassing the pre-, peri-, and post-COVID-19 phases. A significant increase in CPE prevalence was observed during the pandemic period, with KPC remaining the most frequently detected carbapenemase—ranging from 39.9% to 53.5% annually. Additionally, the study identified a rapid transition from VIM to NDM-type metallo-β-lactamases, indicating a fundamental shift in the molecular epidemiology of CREs in this setting. Furthermore, antimicrobial resistance rates—especially to last-resort agents—significantly increased in 2022 and 2023 compared to pre-pandemic levels, underscoring the clinical impact of this evolving resistance landscape [57].

In Argentina, the RECAPT-AR study found a post-COVID rise in NDM (42%) and KPC (39.8%) among *Enterobacterales*, with frequent co-production of resistance genes in *K. pneumoniae*, highlighting the role of MGEs in facilitating rapid resistance spread [58]. The ECDC reported a rise in carbapenem-resistant *Klebsiella pneumoniae* across EU member states, particularly in bloodstream infections. High-risk clones, including hypervirulent ST23 strains carrying carbapenemase genes, have emerged and spread widely, prompting calls for urgent enhancement of infection prevention, control, and antimicrobial stewardship measures across healthcare systems [20].

The emergence of strains harboring multiple carbapenemases simultaneously is particularly concerning. A multicenter Italian study identified *Enterobacterales* carrying dual or triple carbapenemases, including combinations such as KPC+NDM and NDM+OXA-48, which severely limit available treatment options even with novel agents such as ceftazidime–avibactam and cefiderocol [59]. Similar trends were documented in Croatia, where clinical isolates of *K. pneumoniae* and *E. cloacae* carried OXA-48+NDM, OXA-48+KPC, or OXA-48+VIM, many with extensively drug-resistant (XDR) phenotypes and first reports of mcr genes conferring colistin resistance [60].

At the global level, genomic studies confirmed the rapid dissemination of KPC+NDM co-producing *Enterobacterales*, a phenomenon significantly accelerated post-COVID-19. These strains, frequently associated with multireplicon plasmids, demonstrated pan-β-lactam resistance and marked limitations in therapeutic options [61]. Importantly, Gao et al. demonstrated the efficient transferability of KPC-2 and NDM-1 plasmids in *K. pneumoniae*, highlighting the high potential for horizontal gene transfer in clinical settings [62].

### 5.2. Enzymatic and Non-Enzymatic Mechanisms of Resistance

Enzymatic resistance through carbapenemases was complemented by non-enzymatic mechanisms which intensified under the selective pressures imposed during the COVID-19 pandemic. Porin alterations (e.g., OmpK35/36 in *K. pneumoniae*, OprD in *Pseudomonas aeruginosa*) and efflux pump overexpression (e.g., MexAB-OprM) reduced carbapenem susceptibility and enhanced multidrug resistance profiles [63,64,65,66].

MGEs, particularly high-risk plasmids (IncX3, IncFIIK, IncL/M), were critical in disseminating carbapenemase and extended-spectrum β-lactamase (ESBL) genes, often in association with aminoglycoside resistance genes (rmt, aac), fluoroquinolone resistance (qnr), sulfonamide (sul1/2), trimethoprim (dfrA), and mcr genes conferring colistin resistance [59,60,67]. Under pandemic conditions—characterized by overwhelmed hospital settings, high patient turnover, and widespread use of broad-spectrum antibiotics—these mobile elements facilitated rapid horizontal gene transfer fostering the emergence of multidrug and extensively drug-resistant (MDR/XDR) clones [68].

Taken together, the evidence suggests that the COVID-19 pandemic not only increased the prevalence of CROs but also accelerated the convergence of multiple resistance mechanisms. The emergence of dual or triple carbapenemase producers, plasmid-mediated dissemination of resistance determinants, and the spread of pan-β-lactam resistant lineages represent critical challenges for diagnostics, therapeutics, and containment strategies in the post-pandemic era [59,60,61,62,69,70,71].

### 5.3. Biofilm Formation in COVID-19 Patients

COVID-19 patients—particularly those requiring invasive respiratory support—were frequently colonized by biofilm-forming *Acinetobacter baumannii*, *Pseudomonas aeruginosa*, and *Enterobacterales*, including *Escherichia coli* and *Klebsiella pneumoniae* harboring NDM or IMP carbapenemases [72,73,74]. These biofilms not only exhibited extensive drug resistance but also contributed to persistent environmental contamination in ICUs. A Brazilian study highlighted the emergence of highly virulent, biofilm-producing CRAB strains among critically ill COVID-19 patients, underscoring the dual challenge of antimicrobial resistance and biofilm-mediated persistence in healthcare settings [75]. Biofilms on endotracheal tubes, catheters, and other invasive devices acted as protected reservoirs for multidrug-resistant organisms, facilitating colonization and increasing the risk of ventilator-associated pneumonia (VAP) [76]. Additionally, biofilms may harbor occult SARS-CoV-2, potentially explaining prolonged viral positivity, lung cavitation, and re-positive cases in ICU patients [77]. *Enterobacterales* biofilms were also shown to upregulate genes involved in resistance, adhesion, and virulence, promoting persistence in both clinical and environmental niches [74]. Patients on prolonged mechanical ventilation—particularly those receiving systemic corticosteroids, ECMO, or broad-spectrum antibiotics—displayed heightened susceptibility to colonization and invasive infection by carbapenem-resistant organisms, including CRAB and CRE [78].

### 5.4. Environmental Reservoirs and Wastewater Surveillance

The pandemic also influenced resistance dynamics beyond direct patient care, with both environmental reservoirs and hospital infrastructures acting as amplifiers of resistant organisms. Outbreak investigations demonstrated that OXA-23–producing CRAB strains spread via high-touch surfaces, shared medical equipment, and sinks in ICUs, highlighting vulnerabilities in infection prevention under pandemic stress and overburdened staffing [79,80,81]. Even targeted interventions such as enhanced environmental cleaning proved insufficient to fully prevent CRAB colonization, as demonstrated in Korean ICU settings, where persistent surface contamination sustained transmission despite intensified hygiene measures [82]. Biofilm formation further enhanced environmental persistence, contributing to sustained ICU transmission even under strengthened cleaning protocols [72,76].

Beyond clinical wards, hospital wastewater studies revealed increased abundance and seasonal fluctuation of key resistance genes (e.g., bla_OXA, bla_NDM, bla_KPC, qnrS), despite reduced overall antibiotic concentrations. These genes, often plasmid-associated, were frequently detected in *K. pneumoniae*, *E. coli*, and *A. baumannii*, underscoring the role of effluents as reservoirs and dissemination points for AMR during the pandemic [83]. A meta-analysis of hospital wastewater confirmed persistently elevated levels of antibiotic resistance genes (ARGs)—including carbapenemases such as KPC and NDM—reinforcing wastewater’s critical role in AMR dissemination [84]. At the broader environmental level, municipal wastewater analyses during COVID-19 surges revealed alarming concentrations of antibiotic residues (e.g., azithromycin, cefixime) and ARGs, such as bla_OXA, aadA, cat, and aph3, linked to MGEs, raising concerns about horizontal gene transfer beyond hospitals [85].

Together, these findings underscore the interplay between biofilm formation, gene expression changes, environmental persistence, and horizontal gene transfer in driving the dissemination of CROs during the COVID-19 pandemic.

## 6. Specific Pathogens of Concern

During the COVID-19 pandemic, certain carbapenem-resistant Gram-negative bacteria became increasingly prevalent in HAIs. The most problematic pathogens include *Klebsiella pneumoniae*, *Acinetobacter baumannii*, and *Pseudomonas aeruginosa*—all of which are classified as “critical priority pathogens” by the World Health Organization [1]. These organisms were frequently isolated in ICUs, especially from ventilated patients and those with prolonged hospital stays.

### 6.1. Carbapenem-Resistant Klebsiella Pneumoniae (CRKP)

*K. pneumoniae* was one of the most frequently reported carbapenem-resistant pathogens during the pandemic, especially in countries with high baseline rates of resistance. The COVID-19 ICU environment facilitated the rapid spread of CRKP, particularly strains producing NDM and OXA-48 carbapenemases [57,86]. An ICU outbreak in Italy underscored this trend, linking CRKP spread to invasive devices such as femoral hemodialysis catheters, which were significantly associated with infection risk [87]. Among KPC-producing strains, only approximately 15% remained susceptible to amikacin, and 8% to colistin; NDM producers revealed even lower susceptibility rates [88].

### 6.2. Carbapenem-Resistant Acinetobacter Baumannii (CRAB)

CRAB emerged as a major cause of VAP and bloodstream infections in COVID-19 ICUs. Its ability to persist in hospital environments and survive on surfaces for prolonged periods made it particularly difficult to control during the pandemic [75]. CRAB isolates from COVID-19 units often harbored OXA-23, OXA-91, and NDM-1 genes, showing resistance to nearly all available agents, including colistin, and were linked to biofilm formation and environmental persistence [88]. Investigations of ICU outbreaks further revealed transmission of OXA-23–producing CRAB via shared surfaces, highlighting critical infection prevention failures under pandemic stress conditions [89].

During the COVID-19 pandemic, multiple studies reported significant increases in CRAB infections and colonization. In the United States, hospital-acquired CRAB infections rose by 78% from 2019 to 2020, with an estimated 7500 cases per year resulting in approximately 700 deaths [8]. In Europe, bloodstream infections caused by *Acinetobacter* spp. increased by 57%, with carbapenem resistance rates climbing from 48.4% in 2018–2019 to 65.8% in 2020–2021 [38,90]. Countries with high pre-pandemic resistance levels experienced a combined 116% increase in reported cases. Other reports indicated smaller but still notable rises ranging from 1.5% to over 620% in specific ICU settings [91]. Genomic surveillance further revealed that CRAB became endemic in ICUs during the pandemic, with evidence of clonal diversification and persistence despite infection prevention measures. A detailed prospective study in the West Pacific region demonstrated that ICU-adapted CRAB lineages not only persisted but also diversified under selective pressures, reinforcing the difficulty of eradication once established [92]. These outbreaks frequently occurred in ICUs where infection prevention measures were disrupted, resources were diverted, or antimicrobial stewardship programs were deprioritized, creating an environment highly conducive to CRAB transmission [6].

### 6.3. Carbapenem-Resistant Pseudomonas Aeruginosa (CRPA)

CRPA was another key pathogen of concern during the pandemic, particularly in Latin America, Europe, and the Middle East [43]. CRPA infections in COVID-19 patients were often associated with poor outcomes due to limited treatment options and delayed diagnosis. As reported by the ATLAS surveillance program, which analyzed over 30,500 *Pseudomonas aeruginosa* isolates from 157 medical centers across six global regions (2018–2022), CRPA rates remained relatively stable over the pandemic period, despite rising DTR phenotypes in all regions except North America [41]. Notably, most CRPA isolates lacked carbapenemase genes, underscoring the dominance of non-enzymatic mechanisms in *P. aeruginosa* [43]. Complementing these observations, the multicountry prospective cohort study reported substantial mortality among CRPA infections, particularly those caused by metallo-β-lactamase–producing strains (NDM, VIM, IMP), while also documenting clear regional variation in carbapenemase distribution [93]. These findings highlight how the pandemic exacerbated the clinical impact of CRPA, not only by driving selective pressure for resistance but also by amplifying poor patient outcomes in settings with limited therapeutic options.

## 7. Treatment of Carbapenem-Resistant Bacteria During the COVID-19 Pandemic

The management of carbapenem-resistant infections in COVID-19 patients has been particularly challenging, given the high prevalence of co-infections, increased ICU stays, and limited therapeutic options. Combination therapy emerged as the most widely applied approach, often involving polymyxins, tigecycline, fosfomycin, aminoglycosides, and newer β-lactam/β-lactamase inhibitor combinations, depending on availability and local resistance profiles.

### 7.1. Antimicrobial Therapy

Notably, recent studies have demonstrated that early initiation of multidrug regimens may improve outcomes in critically ill COVID-19 patients with CRAB. For example, Heil et al. reported that early initiation of three-drug combinations, including agents such as colistin, tigecycline, and fosfomycin, was associated with more favorable microbiological and clinical outcomes compared with delayed or monotherapy approaches [94]. A retrospective study across four Italian hospitals assessed the efficacy of cefiderocol, a novel siderophore cephalosporin, in treating severe CRAB infections in COVID-19 patients [95]. The study found that cefiderocol monotherapy was associated with improved 28-day survival rates compared to best available therapy, suggesting its potential role in managing such infections.

For CRKP, combination therapy was often employed [96]. A study in a Greek tertiary hospital during the pandemic evaluated the impact of a carbapenem-focused antimicrobial stewardship program. The program led to a reduction in carbapenem consumption and improved patient outcomes, highlighting the importance of targeted antimicrobial stewardship in managing resistant infections [97].

### 7.2. Infection Control and Stewardship

The pandemic underscored the critical need for robust infection control measures and antimicrobial stewardship programs. A multicenter study in Italy observed a significant increase in colonization and infection rates with carbapenem-resistant bacteria during the COVID-19 pandemic. The implementation of enhanced infection control practices and stewardship interventions was essential in mitigating the spread of these resistant pathogens [98].

### 7.3. Challenges and Future Directions

Despite these efforts, challenges remain in the management of carbapenem-resistant infections. The emergence of resistance to novel agents like cefiderocol and the limited availability of alternative therapies necessitate ongoing research and development [95]. Additionally, the disparity in access to effective antimicrobials, particularly in LMICs, exacerbates the global burden of AMR.

## 8. Mitigation Strategies for Carbapenem Resistance in the Post-Pandemic Era

The COVID-19 pandemic has underscored the urgent need to reinforce global responses to AMR, particularly against CROs. Excessive empirical use of broad-spectrum antibiotics, including carbapenems, created additional selection pressure [99], while the breakdown of established IPC protocols contributed to outbreaks in ICUs worldwide [39,100]. As healthcare systems recover, ASPs must be re-established and resourced, integrating digital tools and tele-stewardship approaches [101]. Importantly, stewardship showed adaptability through digital platforms: tele-stewardship and remote decision-support systems sustained oversight of antimicrobial use when in-person consultations were limited, reducing inappropriate prescribing and extending ASP reach. Multiple studies confirmed that telemedicine-supported stewardship interventions during COVID-19 were feasible and effective in maintaining safe medication practices across diverse clinical settings [102,103,104]. Complementing stewardship, ICU-focused containment strategies have proven successful even under pandemic stress; for instance, Li et al. described how targeted isolation, reinforced screening, and strict adherence to IPC bundles enabled successful control of a CRKP outbreak in a Chinese ICU, demonstrating that rigorous local interventions can mitigate spread even in resource-constrained contexts [105]. These experiences highlight the importance of embedding tele-stewardship into routine practice, particularly in resource-limited or geographically dispersed healthcare systems. IPC measures—including strict adherence to hand hygiene, isolation precautions, and device-associated bundles—remain the cornerstone of resistance containment, complemented by targeted screening of high-risk patients and rapid diagnostics [106]. Investments in laboratory capacity are particularly critical in LMICs, where surveillance and diagnostic gaps delayed outbreak recognition [42,107]. Rapid molecular diagnostics paired with ASPs have been shown to improve time-to-therapy and reduce mortality in bloodstream infections, underscoring their value for early detection and control [108,109]. Strengthened global surveillance, through systems such as WHO GLASS, and better cross-border data sharing will be essential to monitor resistance trends and prevent regional spread [42].

Beyond stewardship and IPC, addressing carbapenem resistance requires renewed investment in therapeutics and innovation. Despite recent advances, such as cefiderocol, sulbactam–durlobactam, and other novel β-lactam/β-lactamase inhibitor combinations [110,111,112], access to these agents remains highly uneven, with marked disparities between high-income countries and LMICs [42,113,114]. Global initiatives like the AMR Benchmark and GARDP emphasize the need for policies that promote equitable access while sustaining drug development pipelines [113,114]. Artificial intelligence (AI) and machine learning (ML) represent promising adjuncts to antimicrobial stewardship and surveillance; however, their real-world effectiveness remains unproven [115]. While ML models have demonstrated the ability to guide empirical therapy in ICU settings [116,117], predict resistance patterns earlier than traditional methods [118], and support digital stewardship platforms with high predictive accuracy in retrospective or simulated datasets, these approaches have not yet been fully validated or widely implemented in routine clinical practice [119]. Moreover, barriers remain, including limited data standardization, clinician trust, and infrastructure in resource-constrained settings [120]. Together, these mitigation strategies—summarized in Table 1—highlight the importance of embedding resilience, innovation, and equitable access into AMR control efforts to reverse pandemic-era setbacks.

## 9. Conclusions

The COVID-19 pandemic significantly altered the landscape of AMR, particularly with regard to carbapenem-resistant organisms. A convergence of factors—including increased empiric and often unnecessary antibiotic use, disruption of antimicrobial stewardship programs, and weakened infection prevention efforts—created ideal conditions for the spread of carbapenem-resistant pathogens.

While these effects were more pronounced in healthcare systems with limited resources or high baseline AMR burdens, even high-income countries reported alarming trends in carbapenem resistance during the pandemic. The global rise in resistance underscores the fragility of prior progress and the urgent need to implement cross-cutting reforms. The lessons learned from COVID-19 should stimulate international efforts to address the AMR crisis—not as a parallel challenge, but as an integral part of public health emergency preparedness.

## Figures and Tables

**Table 1:** Summary of Mitigation Strategies Against Carbapenem Resistance in the Post-COVID-19 Era.

Strategy	Objective	Key Actions
Infection Prevention and Control (IPC)	Reduce transmission of resistant organisms in healthcare settings	- Reinforce hand hygiene and contact precautions - Improve environmental cleaning - Cohorting of patients
Antimicrobial Stewardship Programs (ASPs)	Optimize antibiotic prescribing and reduce unnecessary carbapenem use	- Reintroduce ASP audits and feedback - Promote guideline-based empiric therapy - Educate clinicians in antibiotic prescribing - Prompt antibiotics de-escalation
Laboratory and Surveillance Capacity	Detect and monitor resistance trends	- Extend carbapenemase detection - Implement real-time reporting systems - Join national AMR networks
Novel Antibiotics	Provide effective alternatives to carbapenems	- Use new β-lactam/β-lactamase inhibitors - Ensure stewardship supervision
Global Policy and One Health	Coordinate efforts across human, animal, and environmental health	- Regulate antibiotic use in agriculture - Address environmental contamination - Promote international collaboration
AI and Machine Learning Integration	Support diagnostics, surveillance, and stewardship	- Predict resistance risk/outbreaks - Support empiric therapy decisions - Automate data analysis and alerts

## Data Availability

No new data were created or analyzed in this study. Data sharing is not applicable to this article.
